# Safety and Efficacy of Upadacitinib in Patients with Inflammatory Bowel Disease After Liver Transplantation: A Case Series

**DOI:** 10.1007/s10620-025-09569-9

**Published:** 2025-11-20

**Authors:** Yusuke Miyatani, David Choi, Andrew I. Aronsohn, Anjana Pillai, Russell D. Cohen, David T. Rubin

**Affiliations:** 1 Department of Medicine, University of Chicago Medicine Inflammatory Bowel Disease Center, 5841 S. Maryland Avenue, MC 4076, Chicago, IL 60637 USA; 2 Center for Liver Diseases, University of Chicago Medicine, Chicago, IL USA

**Keywords:** Upadacitinib, Janus kinase inhibitors, Post-liver transplant, Inflammatory bowel disease

## Abstract

**Purpose:**

Approximately 2% of patients with inflammatory bowel disease (IBD) have primary sclerosing cholangitis (PSC), and some require liver transplantation (LT). Managing IBD after LT is challenging given concomitant anti-rejection immunotherapies. We report our experience using upadacitinib (UPA) to treat patients with IBD after LT.

**Methods:**

Retrospective, single-center observational study at a tertiary center, identifying patients after LT who received UPA. We assessed efficacy and safety of UPA.

**Results:**

Four patients after LT (Crohn’s disease *n* = 3; ulcerative colitis *n* = 1) received UPA for IBD control (*n* = 3) or as a steroid-sparing adjunct for anti-rejection (*n* = 1), alongside anti-rejection immunosuppression. Median follow-up from UPA initiation was 10.5 months (IQR 8.9–14.6); age 41.5 years (IQR 40–44); interval from LT 3.2 years (IQR 2.3–5.6). Two receiving prednisone for Crohn’s control at baseline achieved steroid-free remission (Harvey–Bradshaw Index < 5). Three developed liver enzyme elevation: one stopped UPA at one month with subsequent normalization of alanine and aspartate aminotransferase; one underwent liver biopsy showing no rejection and continued UPA with 9-month follow-up; and one receiving UPA for potential anti-organ rejection plus vedolizumab ultimately discontinued UPA for suspected rejection after tapering steroids. One patient experienced mild COVID-19 that resolved without treatment change. No life-threatening adverse events were observed.

**Conclusion:**

In this small series, UPA controlled IBD activity in 2 of 4 patients after LT but was associated with liver-enzyme elevations in 3, prompting discontinuation in 2. These findings support cautious, closely monitored use and highlight the need for a larger multi-center study of UPA in patients with IBD after LT.

## Introduction

Primary sclerosing cholangitis (PSC) is a well-known extraintestinal manifestation of inflammatory bowel disease (IBD), characterized by chronic inflammation and fibrosis of bile ducts [1, 2]. Approximately 2% of patients with IBD develop PSC, whereas up to 80% of patients with PSC have concurrent IBD [3, 4]. PSC can progress to advanced cirrhosis, cholangiocarcinoma, and subsequently require liver transplantation (LT) within 15–20 years of diagnosis [5]. On the other hand, the disease activity of IBD with concomitant PSC is generally quiescent with subclinical or milder inflammation [5]. However, some studies showed that the disease progression of IBD can become more aggressive after LT regardless of concomitant anti-rejection immunotherapies, which makes treating IBD challenging [6]. The utility and safety profile of anti-tumor necrosis factor-α inhibitor and anti-α4β7 integrin were reported in the real-world data for patients with IBD after LT [7]. However, efficacy and safety data of newer advanced therapies, especially Janus kinase inhibitors (JAKi), remain scarce.

Upadacitinib (UPA), a selective JAKi, is a novel small molecule for patients with moderately to severely active IBD [8, 9]. JAKi suppresses the JAK/signal transduction and activators of transcription (STAT) signaling pathway, thereby attenuating pro-inflammatory cytokine responses, which could theoretically improve graft survival after organ transplantation [10].

Here, we describe our experience with UPA, a selective Janus kinase-1 inhibitor, for patients with IBD who underwent LT combined with anti-rejection immunosuppression therapy.

## Case Series

### Baseline Characteristics

We identified four patients with IBD after LT who initiated UPA (three ileocolonic Crohn’s disease [CD] and one ulcerative colitis [UC] with ileal pouch anal anastomosis) with a median age of 41.5 years (IQR 40–44) and a median IBD duration of 18.5 years (IQR 11.8–22.5) starting UPA (Table 1). Three were diagnosed with IBD before LT. All patients underwent LT for PSC, and the median interval from the transplant to starting UPA was 3.2 years (IQR 2.3–5.6). Three patients were initiated on UPA for the treatment of active IBD, and one was used as a potential anti-rejection treatment. UPA was initiated, replacing advanced therapy for IBD (risankizumab, adalimumab, and ustekinumab), and vedolizumab (Case 3) was continued in addition to their anti-rejection medications for LT, such as mycophenolate, tacrolimus, and prednisone. No patient had been exposed to JAKi before starting UPA. Two patients were started with an initial dose of UPA of 30 mg/day, and the others with 15 mg/day. Patients were followed up with a median total length from UPA initiation of 10.5 months (IQR 8.9–14.6).

**Table 1:** Baseline characteristics at upadacitinib initiation in patients with IBD after liver transplantation

Case	Age, years	Sex	IBD type	Disease location	IBD duration at UPA start, years	Timing of IBD diagnosis	Prior IBD therapies	Indication for LT	Time from LT to UPA start, years
1	50	F	CD	Ileocolonic	2	Post-LT	5-ASA, pred, IFX, VDZ, UST, RZB	PSC	11.6
2	41	F	CD	Ileocolonic	24	Pre-LT	5-ASA, pred, AZA, IFX, ADA, CER, NAT, VDZ, UST, RZB	PSC	2.8
3	42	M	CD	Ileocolonic	22	Pre-LT	5-ASA, pred, AZA, IFX, ADA, VDZ, UST	PSC	0.9
4	37	M	UC	Pancolitis*	15	Pre-LT	5-ASA, pred, AZA, MTX, IFX, VDZ	PSC	3.7

*IBD* inflammatory bowel disease, *CD* Crohn’s disease, *UC* ulcerative colitis, *LT* liver transplantation, *UPA* upadacitinib, *PSC* primary sclerosing cholangitis, *5-ASA* 5-aminosalicylate, *pred* prednisone, *AZA* azathioprine, *MTX* methotrexate, *IFX* infliximab, *ADA* adalimumab, *UST* ustekinumab, *VDZ* vedolizumab, *RZB* risankizumab, *CER* certolizumab pegol, *NAT* natalizumab

^*^Post proctocolectomy with ileal pouch anal anastomosis

### Efficacy and Safety Outcomes

Two patients (Case 1, 3) who started UPA of 30 mg/day for active IBD achieved steroid-free clinical remission within 12 weeks (Table 2); one (Case 3) attempted tapering UPA from 30 to 15 mg/day and remained in clinical remission for IBD. Three patients (Case 1, 2, 4) developed elevated liver function, two of those required discontinuation of UPA; one (Case 2) showed elevated aspartate aminotransferase (AST)/alanine aminotransferase (ALT) from 14/15 U/L up to 107/57 U/L within a month since starting UPA of 15 mg/day with subsequent AST/ALT normalization; another patient (Case 1) with elevated AST/ALT of up to 83/228 U/L required liver biopsy at 3 months showing nonspecific findings with focal portal inflammation and mild bile duct injury without evidence of rejection. UPA was continued given its significant response to CD, and subsequently, AST/ALT were normalized without changing treatment; the third patient (Case 4), who started UPA as anti-rejection, was on prednisone 10 mg/day, mycophenolate mofetil, and tacrolimus as anti-rejection in addition to vedolizumab every 8 weeks for IBD control. The patient continued all the immunosuppressive medications except for prednisone which was tapered from 10 to 5 mg/day after initiating UPA. The patient developed elevated ALT (41 U/L) and alkaline phosphatase (243 IU/L). Given a prior episode of biopsy-confirmed rejection that presented with ALT and alkaline phosphatase elevations following prednisone taper, allograft rejection was suspected, and UPA was discontinued. No severe infection was observed except for one patient, who acquired a mild case of COVID-19, which resolved with no intervention. No life-threatening adverse events were observed in all patients. All the decisions regarding UPA were made after multidisciplinary discussion between IBD and transplant hepatologists, with risks and benefits explained to the patients.

**Table 2:** Treatments and clinical outcomes during upadacitinib therapy after liver transplantation

Case	Anti-rejection at UPA start	IBD therapy at UPA start	UPA starting dose, mg/day	Efficacy	Rejection	Adverse events	Continue UPA at last follow-up	Reason for discontinuation	UPA exposure, months	Total follow-up time from UPA, months
1	MMF TAC	RZB* pred 20 mg	30	HBI 8 → 3 at week 8; steroid-free	No	Elevated LFTs	Yes	NA	9	9
2	MMF TAC	ADA pred 10 mg	15	NA (early discontinuation)	No	Elevated LFTs	No	Elevated LFTs	1.5	22.5
3	TAC	UST* pred 30 mg	30	HBI 14 → 1 at week 12; steroid-free	No	None	Yes	NA	12	12
4	MMF TAC pred 10 mg	VDZ	15	pred 10 mg → 5 mg for anti-rejection	Suspected	Elevated LFTs; COVID-19	No	Suspected rejection	5.5	8.5

*UPA* upadacitinib, *MMF* mycophenolate mofetil, *TAC* tacrolimus, *RZB* risankizumab, *ADA* adalimumab, *UST* ustekinumab, *VDZ* vedolizumab, *pred* prednisone, *HBI* Harvey–Bradshaw Index, *LFTs*, liver function tests, *COVID-19* coronavirus disease 2019

^*^Medication was replaced with UPA

## Discussion

Our case series highlights a potential hepatic safety signal with UPA in post-LT IBD with evidence of clinical benefit for IBD control. Three out of four patients (75%) developed elevated liver enzymes after UPA initiation: two required discontinuation of UPA within 6 months based on the clinical decision, and one continued after liver biopsy excluded organ rejection following multidisciplinary review.

In the phase 3 maintenance trial of UPA and long-term safety data of tofacitinib (TOF) in patients with IBD, both JAKi showed reversible hepatic adverse events in a dose-dependent manner, specifically mild to moderate transaminase elevations [8, 9, 13]. Several mechanisms have been proposed. UPA can plausibly raise transaminase by attenuating hepatocellular IL-6/gp130/STAT3 signaling, a pathway that supports cytoprotection and regeneration during liver injury [14]. Inhibiting these protective signals makes hepatocytes more susceptible to injury from intercurrent hits, including viral infection, ischemia, or concomitant hepatotoxins, manifesting more readily as enzyme elevations. In addition, pharmacokinetic factors may also contribute. An open-label, single-dose study enrolling participants with Child–Pugh A, B cirrhosis, and those without cirrhosis was conducted to evaluate the pharmacokinetics of UPA using a 15-mg extended-release formulation [15]. The central ratios of UPA maximum observed concentration within 7 days in the participants with Child–Pugh B were 43% higher compared with those without cirrhosis. While the study did not show a statistical difference, likely attributed to a small sample size (*n* = 6 in each group) and lower dose of UPA, a change in cytochrome P450 3A4 activity, which metabolizes UPA, in the impaired hepatic function group, may have contributed to the pharmacokinetic effect. Further exploration is needed to elucidate the exact mechanism for hepatic events due to JAKi use in patients after LT.

In real-world data, only one case report and case series regarding JAKi use in patients with IBD after LT are available [16, 17]. In a previous case report, a patient with UC started an induction dose of TOF of 10 mg twice a day for refractory UC in addition to his anti-rejection immunosuppression 15 years after LT. In a 3-month follow-up period, the patient achieved clinical and endoscopic remission in UC, and continued TOF with a maintenance dose of 10 mg/day without hepatic adverse effects [16]. The other case series included four patients with IBD after LT for PSC who initiated UPA with an induction dose of 45 mg/day followed by a maintenance dose of 30 mg/day (UC, *n* = 2; CD, *n* = 1; pouchitis, *n* = 1) [17]. Of note, three of four patients in the cohort were switched from TOF to UPA due to treatment failure for IBD, not due to liver-related adverse events. Nevertheless, all the patients achieved clinical remission after starting UPA with a median duration of 9.5 months (IQR 7–11.8). Notably, there was no patient who developed liver toxicity in both studies, regardless of the higher induction therapy of JAKi. This discrepancy in the results may be partially attributed to the selection bias of known liver tolerance to TOF, as all the patients in our cohort did not have previous exposure to TOF, which highlights the further need to explore the best usage of UPA in post-LT.

For one patient in our cohort, UPA was added to existing immunosuppressive therapy as a potential steroid-sparing agent after multidisciplinary discussion between IBD and transplant hepatology. Unfortunately, our patient had clinically suspected rejection after tapering down prednisone, even with this addition, which ultimately led to discontinuation of UPA. While no human data on UPA as an anti-rejection therapy in post-solid organ transplantation are available, there is some evidence showing the benefit of TOF to prevent organ rejection, especially in the kidney transplant field [18]. Long-term extension of Phase 2 b randomized trial compared TOF with cyclosporine for patients after kidney transplant showed a lower rate of renal rejection, preserving renal function. However, serious infection was observed in patients treated with TOF in a dose-dependent manner.

Although there were some patients who developed elevated liver enzymes with UPA in our study, it is important to consider the other differentials of liver enzyme elevations among patients after LT, including infection, recurrent PSC, biliary or vascular complications [11, 12]. Utilizing a multidisciplinary approach to initiating and stopping UPA is key to ensuring successful initiation and monitoring of therapy.

There are several limitations to this case series. First, the sample size (*n* = 4) precludes statistical inference and limits generalizability. A larger, multi-center study is needed to confirm these findings. Second, the follow-up period from UPA initiation was relatively short, which may have underestimated long-term adverse events such as malignancies. Third, as a retrospective, single-center case series without a comparator, our data on efficacy and safety are vulnerable to selection and information bias. Despite these limitations, reporting these cases adds value given the paucity of evidence of UPA after LT in patients with IBD, and potential hepatic safety signals that have not been highlighted in prior reports.

In conclusion, UPA may be a therapeutic option for the treatment of IBD in patients after LT, but careful monitoring for hepatic adverse events is essential. Further multi-center studies are needed to define its long-term safety and to optimize its use in this setting, including the timing of initiation and dosing.

## Data Availability

The data from this analysis are available within the article. Any additional data will be shared by the corresponding author upon reasonable request.
